# Bridging Psychiatry and Neurology: A Case of Misdiagnosed Catatonia in Anti-NMDA Receptor Encephalitis

**DOI:** 10.1192/j.eurpsy.2026.11611

**Published:** 2026-07-31

**Authors:** A. Šarskutė - Burneikė, J. Čiuderė, G. Šarskutė

**Affiliations:** 1 Intensive Care Unit, Republican Vilnius Psychiatric Hospital; 2 Intensive Care Unite, Republican Vilnius Psychiatric Hospital, Vilnius University; 3 Vilnius University, Vilnius, Lithuania

## Abstract

**Introduction:**

Anti-NMDA receptor encephalitis (ANMDARE) is a rare, rapidly progressive autoimmune disease of the central nervous system that may cause severe neurological deficits or even death. The initial phase is often dominated by psychiatric symptoms, followed by neurological and autonomic manifestations. The disorder predominantly affects young women. Catatonic syndrome may be misinterpreted as a manifestation of non-organic psychosis, complicating timely diagnosis and leading to a poorer prognosis.

**Objectives:**

To present a case of ANMDARE initially manifested by catatonic and acute psychiatric symptoms, emphasising diagnostic challenges, treatment complications, and the importance of interdisciplinary management

**Methods:**

A 35-year-old woman with no identifiable psychosocial stressors developed behavioural changes, mood fluctuations, severe anxiety, insomnia, and transient confusion. Catatonic symptoms gradually emerged, and neurological pathology was initially excluded. Despite 21 days of high-dose neuroleptic treatment exceeding therapeutic limits, her condition progressed to catatonic stupor. She was referred for electroconvulsive therapy (ECT), which was withheld. The clinical picture further worsened with the development of sopor, pulmonary embolism, severe pneumonia, and dysphagia. As the condition continued to deteriorate, the patient was transferred from the psychiatric hospital intensive care unit (ICU) to a general hospital ICU due to clinical suspicion of ANMDARE. The diagnosis was subsequently confirmed based on the clinical presentation and investigations (positive anti-NMDAR antibodies, EEG showing reduced cerebral reactivity, and normal brain MRI).

**Results:**

Prolonged neuroleptic therapy significantly aggravated the clinical condition, intensifying catatonic features, impairing consciousness and inducing extrapyramidal symptoms. The patient was treated for 41 days in the ICU, including 22 days of invasive mechanical ventilation. Specific therapy was administered: plasma exchange, methylprednisolone and subsequent biological treatment. A positive clinical response was achieved. Recovery took up to one year, during which the patient relearned to speak and walk.

**Conclusions:**

ANMDARE may initially present solely with psychiatric manifestations, making early diagnosis difficult. Misinterpretation as a primary psychotic disorder may result in inappropriate neuroleptic therapy or ECT consideration, worsening the patient’s condition and prognosis. This case highlights the need for an interdisciplinary approach involving psychiatrists, neurologists, immunologists and intensivists to ensure accurate diagnosis, timely treatment and improved clinical outcomes.

**Disclosure of Interest:**

None Declared

